# Blocking MIF secretion enhances CAR T-cell efficacy against neuroblastoma

**DOI:** 10.1016/j.ejca.2025.115263

**Published:** 2025-03-11

**Authors:** Josephine G.M. Strijker, Guillem Pascual-Pasto, Grant P. Grothusen, Yannine J. Kalmeijer, Elisavet Kalaitsidou, Chunlong Zhao, Brendan McIntyre, Stephanie Matlaga, Lindy L. Visser, Marta Barisa, Courtney Himsworth, Rivani Shah, Henrike Muller, Linda G. Schild, Peter G. Hains, Qing Zhong, Roger R. Reddel, Phillip J. Robinson, Xavier Catena, María S. Soengas, Thanasis Margaritis, Frank J. Dekker, John Anderson, Jan J. Molenaar, Kristopher R. Bosse, Wei Wu, Judith Wienke

**Affiliations:** a Princess Máxima Center for Pediatric Oncology, Utrecht, the Netherlands; b Division of Oncology and Center for Childhood Cancer Research, Children’s Hospital of Philadelphia; Philadelphia, PA 19104, USA; c Singapore Immunology Network (SIgN), Agency for Science, Technology and Research (A⁎STAR), 8A Biomedical Grove, Immunos, Singapore 138648, Singapore; d Department of Pharmacy and Pharmaceutical Sciences, National University of Singapore, Singapore 117543, Singapore; e Department of Chemical and Pharmaceutical Biology, Groningen, Research Institute of Pharmacy (GRIP), University of Groningen, Antonius Deusinglaan 1, Groningen 9713 AV, the Netherlands; f UCL Great Ormond St Institute of Child Health, London, UK; g ProCan, Children’s Medical Research Institute, The University of Sydney, Westmead, NSW, Australia; h Melanoma Laboratory, Molecular Oncology Programme, Spanish National Cancer Research Centre (CNIO), Madrid, Spain; i Department of Pharmaceutical Sciences, University Utrecht, Utrecht, the Netherlands; j Department of Pediatrics, Perelman School of Medicine at the University of Pennsylvania, Philadelphia, PA 19104, USA; k Biomolecular Mass Spectrometry and Proteomics, Bijvoet Center for Biomolecular Research and Utrecht Institute for Pharmaceutical Sciences, Utrecht University, Utrecht, the Netherlands

**Keywords:** Immunosuppressive tumor microenvironment, CAR T-cell therapy, Neuroblastoma, MIF, PROTAC

## Abstract

**Introduction:**

Chimeric antigen receptor (CAR) T-cell therapy is a promising and innovative cancer therapy. However, immunosuppressive tumor microenvironments (TME) limit T cell persistence and durable efficacy. Here, we aimed to identify and target immunosuppressive factors in the TME of neuroblastoma, a pediatric extracranial solid tumor, to improve CAR-T efficacy.

**Methods:**

Immunosuppressive factors were identified using a multi-omics approach, including single-cell RNA sequencing (scRNA-seq) of 24 neuroblastoma tumors, published bulk-RNA sequencing datasets, and mass-spectrometry of patient-derived tumoroid models. Candidate targets were validated with functional assays *in vitro* and *in vivo*. Protein degradation of the top immunosuppressive target by PROTAC technology was used to evaluate the effect on CAR T-cell activity.

**Results:**

ScRNA-seq revealed 13 immunosuppressive interactions in the TME of neuroblastoma, two effectors of which, Midkine (MDK) and Macrophage Migration Inhibitory Factor (MIF), were validated as candidate targets across multiple published datasets. Both factors were among the top 6 % of most abundantly secreted factors by patient-derived tumoroid models, substantiating their potential relevance in the TME. *In vitro* and *in vivo* functional assays confirmed MIF to be a potent inhibitor of CAR T-cell activation and killing capacity. To translate these findings into a potentially clinically applicable treatment, we explored MIF targeting by PROTAC technology, which significantly enhanced activation of CAR T-cells targeting GPC2 and B7-H3.

**Conclusion:**

By defining the immunosuppressive effects of neuroblastoma’s TME on CAR T-cell efficacy, revealing the pivotal role of MIF, we provide an analytic pipeline and therapeutic strategy for improving adoptive cell therapies for this pediatric malignancy and potentially other solid tumors.

## Highlights

• Inhibitory factors challenge optimal efficacy of CAR-T cell therapy in solid tumors.
• Multi-omics identified MIF and MDK as immunosuppressive targets in neuroblastoma.
• Knock-out of MIF in neuroblastoma increased CAR-T cell efficacy *in vitro* and *vivo*.
• MIF degradation by PROTAC technology significantly reduced tumoral MIF secretion.
• Combining MIF-PROTAC with CAR-T therapy significantly increased CAR-T activation.

## 1 Introduction

Following major success of immunotherapies for adult cancer patients, these innovative treatments are now entering the stage in pediatric solid cancers [1], [2]. Adoptive cell therapy, including chimeric antigen receptor (CAR) T-cell therapy, has led to substantially increased survival rates in multiple cancers and has recently shown early promise in high-risk neuroblastoma, an extracranial solid pediatric cancer with a currently dismal survival rate below 50 % [3], [4].

Several types of CAR T-cells are currently under development for neuroblastoma, both in early phase clinical trials and late-phase preclinical testing. Among these are a second-generation CAR-T targeting the signaling co-receptor glypican-2 (GPC2) and a second-generation CAR-T targeting B7-H3, both well-known tumor antigens expressed by multiple pediatric cancers, including neuroblastoma [5], [6]. While more promising results of CAR T-cell therapy are emerging, immunosuppressive tumor microenvironments (TME) limit optimal persistence and durable clinical effects in solid tumors [7], [8]. In a recent clinical trial with third-generation GD2 targeting CAR T-cells in neuroblastoma, mainly patients with a low tumor burden responded well, suggesting that a higher tumor burden remains a significant challenge for CAR T-cell therapies [9]. Understanding the mechanisms through which solid tumors suppress the optimal activation of CAR T-cells is pivotal to targeting this suppression and advancing CAR T-cell therapy.

The immunosuppressive TME in neuroblastoma encompasses well-known immune checkpoints expressed on the cell membrane, such as PD-L1 and CD200, as well as secreted immunoregulatory mediators, such as TGF-β, galectin-1, and soluble GD2 [10], [11], [12], [13], [14], [15], [16]. These secreted factors can impair the activation of lymphocytes, including CAR T-cells, not only within the TME, but can also reach the systemic circulation, potentially affecting peripheral lymphocytes. Indeed, patients with neuroblastoma often show reduced peripheral T cell counts, and these cells lack responsiveness to *in vitro* activation, indicating a broader T cell dysfunction [17], [18], [19], [20]. This systemic immunosuppression may hinder the long-term persistence, migration and activation of CAR T-cells. However, specific tumor-derived soluble factors that compromise CAR-T efficacy in neuroblastoma remain largely unidentified.

To improve CAR T-cell efficacy, we sought to identify and target immunosuppressive factors secreted by neuroblastoma that may inhibit CAR T-cells and hamper their function locally and/or distally. Using a multi-omics approach combining single-cell RNA sequencing (scRNA-seq) and proteomics, we identified Macrophage Migration Inhibitory Factor (MIF) as a candidate immunosuppressive factor. Reduction of MIF secretion through innovative protein degrader (PROTAC) technology significantly increased CAR T-cell efficacy. Targeting MIF may therefore be a viable strategy to increase the efficacy of CAR T-cell therapy for patients with neuroblastoma.

## 2 Material and methods

### 2.1 Ethical approval

This study complies with all relevant ethical regulations. Studies involving primary tumor or patient data, were performed with data published before according to ethical approval. Primary human T-cells were obtained from anonymous donors through the Human Immunology Core at the University of Pennsylvania under a protocol approved by the CHOP Institutional Review Board. Donors provided informed consent through the University of Pennsylvania Human Immunology Core. Animal experiments were conducted under protocols approved by the CHOP Institutional Animal Care and Use Committee (IACUC; Protocol #1464) with adherence to the NIH guide for the Care and Use of Laboratory Animals accredited by the Association for Assessment and Accreditation of Laboratory Animal Care (AAALAC).

### 2.2 Transcriptomics analyses

Datasets used for transcriptomics analyses were previously published by our and other groups [21], [22], [23], [24]. Cell isolation, library preparation, and cluster annotations are described in the publications. Receptor-ligand interactions between cell subsets in the neuroblastoma TME were predicted using the CellChat algorithm [25], as previously described [21], yielding an unbiased network of ligand-receptor interactions between cell subsets in the TME. Functionally relevant interactions possibly affecting lymphocyte cytotoxicity were selected by correlating the predicted interactions involving CD8^+^ T cells, γδ-T cells and NK cells, with a generated cytotoxicity score of those T/NK cell subsets, which was based on an adapted cytotoxicity score from Tirosh et al. [26], comprising the following genes: *NKG7, CCL5, CST7, PRF1, GZMA, GZMB* and *IFNG*. In short: we overlaid 1) genes B expressed by cell subset X, that had a significant negative correlation (r < 0, p < 0.05) with the cytotoxicity score of T/NK cell subset Y and 2) genes B which were predicted to significantly interact (p < 0.05) in a ligand-receptor pair between cell subset X and T/NK cell subset Y. Only interactions with a significant negative correlation with the cytotoxicity score and predicted interaction with T/NK cells are shown in the figures. Analyses with bulk-RNA transcriptomics were performed on the R2 data platform (R2: Genomics Analysis and Visualization Platform (http://r2.amc.nl)).

### 2.3 Tumoroid cultures for proteomics

Patient-derived tumoroids were previously established and cultured as published before and described in detail in the Supplementary methods [27], [28]. Three representative model for aggressive high-risk neuroblastoma were chosen: two with MCYN amplification (one with 11q loss), and one MYCN non-amplified model with an ATRX homologous deletion (Supplementary Fig. 3b). For whole cell proteomics, one full T175 flask of tumoroids was harvested to make a pellet. Sample preparation and mass spectrometry were performed as described before [29]. For secretome proteomics, empty DMEM GlutaMAX was conditioned on tumoroid cells for 24 h. The conditioned medium was concentrated by filtering through a 3 kDa molecular weight cut-off Millipore filter. Samples were further prepared, measured on LC-MS, and analyzed as described in the Supplementary methods.

### 2.4 Suppression assays

Healthy donor PBMCs were isolated by Ficoll-paque density centrifugation. CD3+ T cells were FACS-sorted and stimulated with α-CD3/α-CD28 Dynabeads (Gibco, cat. 11131D) in the presence of 10 ng/mL recombinant MIF (Biolegend, cat. 599404) or Midkine (MDK) (Biolegend, cat. 754104) for 4 days. Subsequently, cells were washed and stained for flow cytometric analysis. Detailed staining protocol and antibodies are described in the Supplementary methods.

### 2.5 Cell lines and modifications

Neuroblastoma cell lines SK-N-AS, SH-SY5Y, Kelly and SK-N-BE2C were cultured in RPMI 1640 with 10 % FBS, 1 % L-Glutamine and 1 % Penicillin-Streptomycin at 37 °C with 5 % CO_2_. Short-hairpin RNA (shRNA) constructs obtained from Sigma (TRCN0000303918, TRCN0000331210, TRCN0000331252, TRCN0000331270, TRCN0000331211, TRCN0000056818, TRCN0000056819, TRCN0000056820, TRCN0000056821, TRCN0000056822) were used to prepare MIF and MDK knock downs, using lentiviral transduction. Protein expression and concentration was determined using Western Blot and ELISA assays (Supplementary methods).

### 2.6 CAR T-cell manufacturing

GPC2 targeting CAR T-cells were generated as published before and described in the Supplementary methods [30]. After production and expansion, cells were collected, cell viability and CAR expression was determined, and cells were frozen until functional *in vitro* or *in vivo* assays. CD19-targeting CAR-T cells were used as a non-specific control, as CD19 is a B-cell specific molecule and not expressed by neuroblastoma cells [31]. For the production of B7-H3 targeting CAR-T cells, we used previously published TE9–28z CAR-T cells [5], with a similar transduction protocol as published. Virus was produced using Phoenix-Ampho cells, transduced to constitutively express the CAR construct and produce virus. CAR expression was determined on day 3 after transduction by flow cytometry.

### 2.7 Co-culture activation and killing assays

To determine the activation of CAR T-cells in co-culture with MIF or MDK wild-type or knock-down neuroblastoma cells ELISA for IFN-γ and/or flow cytometry for activation markers was used. To determine the killing of tumor cells an IncuCyte readout or a luminescence-based readout was used. Detailed methods for killing assays are described in the Supplementary methods.

### 2.8 Mouse study

To generate SK-N-BE2C wild-type or MIF knock-down xenografts, a total of 5x10^6^ SK-N-BE2C cells were injected subcutaneously in the flanks of 6-week-old immunodeficient female NSG mice (NOD-scid IL2Rgammanull; 005557; Jackson Labs) using 100 µL of Matrigel. Once tumors reached a volume of 0.15–0.3 cm^3^, a total of 5x10^6^ CAR+ T-cells were administered intravenously in 100 µL of PBS. Tumor volumes were measured at least twice weekly and tumor volumes were calculated as volume = ((diameter 1/2 + diameter 2/2)⁄(3 * 0.5236))/100). Mice were treatment naive and maintained in cages of up to 5 mice under barrier conditions with ready access to food and water following IACUC guidelines (Protocol #643). To minimize number of animals used, the control groups were limited to shCTRL + CD19 CAR T-cells, shCTRL + GPC2 CAR T-cells and shMIF + CD19 CAR T-cells. In Progression Free Survival (PFS), each event was the equivalent of a tumor reaching the experimental endpoint, defined as a tumor volume equal or larger than 2 cm^3^ at the last measurement.

### 2.9 Statistics and reproducibility

Statistical tests were performed using GraphPad Prism v10.0.2 software and are specified in the figure legends. A p-value of < 0.05 was considered statistically significant, unless stated otherwise.

## 3 Results

### 3.1 MIF and MDK are negatively associated with lymphocyte cytotoxicity in neuroblastoma

**Fig. 1:**
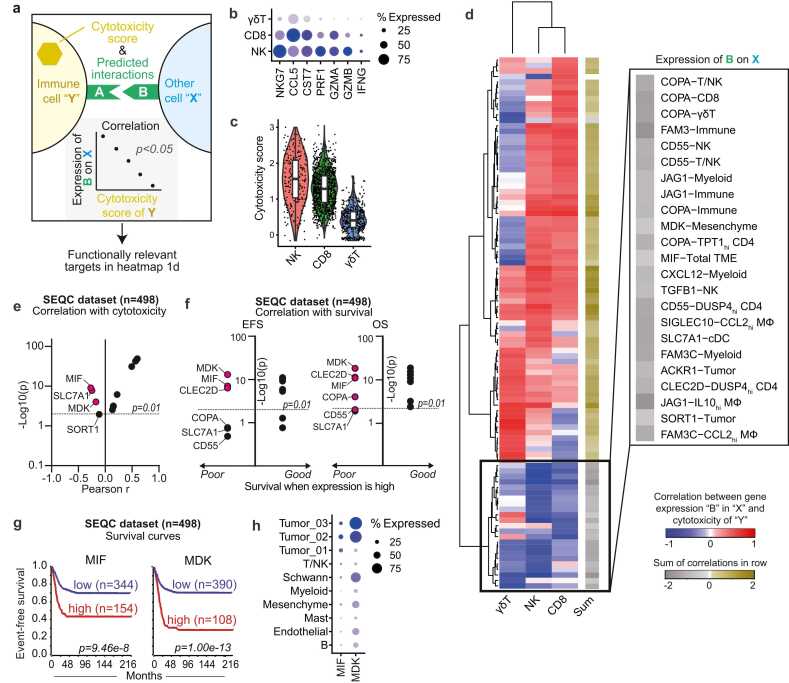
Identification of MIF and MDK as immunosuppressive factors based on transcriptomic data. a, Schematic representation of analysis strategy of scRNAseq data, published earlier by our group, in order to determine significant correlations between the cytotoxicity score of immune cell subsets and predicted interactions of those immune cells with other cells in the tumor microenvironment. b, Dotplot representing the gene expression of 7 cytotoxicity genes to determine the cytotoxicity score of γδ-T cells, CD8 T cells and NK cells. c, Score of cytotoxicity genes in Fig. 1b in NK, CD8-T, and γδ-T cells. *Tukey’s multiple comparisons test for one-way ANOVA.* d, Heatmap representing the correlation between genes in specific cell subsets, which significantly interact with the indicated immune cell subset and significantly correlate with the cytotoxicity of the indicated immune cell subset. Genes with at least one significant correlation, with either NK, CD8-T, or γδ-T, were included. The black box indicates a negative sum of the correlations of the three immune cell subsets and represents the genes selected for further analysis. **e,** Correlation of 13 selected genes from Fig. 1d with cytotoxicity in the dataset with bulk-RNA data from 498 neuroblastoma tumors (r2.amc.nl/; Tumor Neuroblastoma - SEQC - 498 - RPM - seqcnb1; GSE497104243). **f,** Survival analysis using the 13 selected genes from Fig. 1d using the SEQC bulkRNA cohort. Left panel represents event-free survival (EFS) and right panel represents overall survival (OS). Survival when the expression of the gene is high is depicted on the x-axis, the Bonferroni p-value on the y-axis. **g,** Kaplan-Meier curve indicating event-free survival for high- or low expression of *MIF* (left panel, expression cutoff: 157.498) and *MDK* (right panel, expression cutoff: 216.718). Bonferroni p-value is depicted. **h,** Dotplot representing the expression of *MIF* and *MDK* in several cell subsets in scRNAseq dataset [21].

We recently observed, by scRNA-seq of 24 neuroblastoma tumors, that natural killer (NK) cells in the neuroblastoma TME have reduced cytotoxic potential, and that T cells in the same TME display features of dysfunction [21]. To precisely identify which specific immunosuppressive factors in the neuroblastoma TME may be contributing to this reduced NK and T cell cytotoxic potential, we first performed an interaction analysis using CellChat, which predicts interactions between cell subsets based on the expression of ligands and receptors [25]. We focused on interactions of cytotoxic lymphocytes, *i.e.* NK, CD8^+^ T cells, and γδ-T cells, with other cells in the TME. To specifically define which of these predicted interactions may reduce the cytotoxic potential of the immune cell subsets, we analyzed which interactions negatively correlated *(r < 0, p < 0.05)* with the expression of genes encoding cytotoxic mediators (Fig. 1a). To this end, we constructed a cytotoxicity score for each immune cell subset using 7 genes (*NKG7*, *CCL5*, *CST7*, *PRF1*, *GZMA*, *GZMB* and *IFNG),* which is strongly associated with cytotoxicity pathways, as revealed by gene set enrichment analysis (Fig. 1b,c, Supplementary Fig. 1a). Correlation of this cytotoxicity score with the predicted interaction partners yielded 23 interactions involving 13 unique genes, which negatively correlated with NK, CD8^+^ T and/or γδ-T cell cytotoxicity (Fig. 1d). Remarkably, all of these 13 genes were annotated as predicted secreted factors by the SEPDB database of secreted proteins and could thus potentially affect NK/T cells both locally in the TME and distally [32].

(a) Adapted from Wienke et al. [21]. (b) Adapted from Tirosh et al. [26].

To shortlist which of these 13 genes could have the highest potential as a target for combinatorial immunotherapeutic approaches, we validated the correlation between gene expression and immune cell cytotoxicity score in two additional large, independent bulk-RNA sequencing datasets of 498 (SEQC) and 122 (COMBAT-Versteeg) neuroblastoma tumors [22], [23]. Out of the 13 genes, Macrophage Migration Inhibitory Factor (*MIF)* and Midkine (*MDK)* negatively correlated with cytotoxicity in both datasets (Fig. 1e, Supplementary Fig. 1b–e). In addition, high *MIF* and *MDK* expression was significantly associated with inferior event-free and overall survival of neuroblastoma patients, which was independent of MYCN amplification (Fig. 1f, g, Supplementary Fig. 1f–h). The strong consistent correlation with poor cytotoxicity and poor survival led us to focus on MIF and MDK as prime targets to improve the durability of CAR T-cell therapy.

In our scRNA-seq dataset, tumor cells had the highest expression of *MIF* and *MDK*, while other populations, such as monocytes/macrophages, had only low expression, suggesting that tumor cells may be the major source of *MIF* and *MDK* affecting lymphocyte cytotoxicity (Fig. 1h and Supplementary Fig. 1i,j). We confirmed the high expression of *MIF* and *MDK* by tumor cells in another published scRNA-seq dataset of 19 neuroblastoma tumors by Verhoeven et al. [24] (Supplementary Fig. 1k). MIF was predicted to interact with CD8^+^ T cells through the CD74 receptor and MDK with NK cells through the SORL1 receptor, with interactions with tumor cells demonstrating the highest predicted probability (Supplementary Fig. 1l). MIF and MDK are pleiotropic proteins with distinct and context-dependent functions. Both are overexpressed in several cancers and have been previously linked to suppressive effects on T cell cytotoxicity, supporting our observations [33], [34], [35], [36], [37], [38]. Compared to healthy tissues, both *MIF* and *MDK* were overexpressed in neuroblastoma (Supplementary Fig. 2a,b). Moreover, *MIF* was overexpressed in various other pediatric and adult malignancies, suggesting broad relevance as an immunosuppressive target across solid cancers (Supplementary Fig. 2c). *MDK*, however, was less expressed in other cancer types (Supplementary Fig. 2d). Taken together, these data indicate potential immunosuppressive roles of MIF and MDK in the neuroblastoma TME.

### 3.2 MIF and MDK are abundantly secreted by neuroblastoma tumoroids

To validate the potential roles of MIF and MDK in neuroblastoma, we confirmed their protein expression by mass-spectrometry of 11 neuroblastoma tumoroids. MIF was identified among the top 20 most abundant proteins in proteomics of whole tumoroid lysates (Supplementary Fig. 3a). MDK was found in 8 of 11 tumoroids, albeit in lower abundance. However, importantly, considering that both MIF and MDK are secreted proteins, their intracellular levels need not be high for them to mediate extracellular immunosuppressive function.

**Fig. 2:**
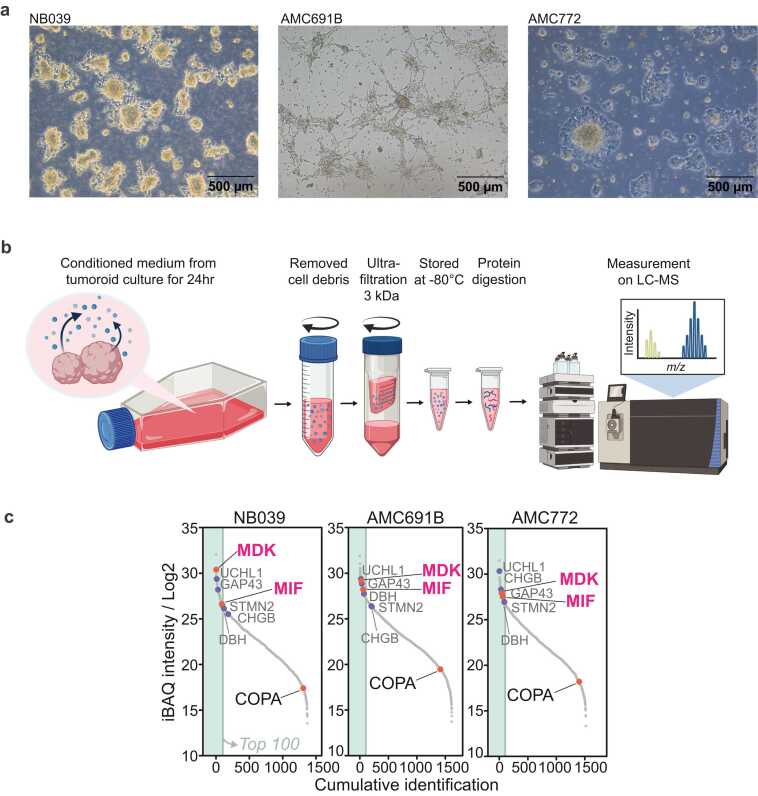
Identification of MIF and MDK in secretome of neuroblastoma tumoroids by LC-MS. a, Pictures of cultures from selected tumoroids for secretome collection. Scalebar indicates 500 µM. b, Schematic overview of procedure to collect and process the secretome for analysis. Further details are provided in the methods section. Figure made with Biorender.com. c, Liquid chromatography–mass spectrometry (LC-MS) analysis of conditioned medium from neuroblastoma tumor organoids detailing their secretome. Red circles/black or pink font: targets identified in Fig. 1d. Blue circles/grey font: neuroblastoma reference proteins. Left green bar indicates top 100 most abundant proteins. iBAQ (intensity Based Absolute Quantification) indicates the protein’s non-normalized intensity divided by the number of measurable peptides indicating the relative abundance of the protein in the sample.

We therefore specifically quantified MIF and MDK secretion by neuroblastoma tumoroids. We selected three representative tumoroid models with distinct genetic backgrounds from which we collected the concentrated secreted proteins (secretome) for analysis by LC-MS (Fig. 2a,b and Supplementary Fig. 3b). Notably, both MIF and MDK were among the top 100 most abundantly secreted proteins in all three tumoroids (Fig. 2c). Taken together, these data firmly establish MIF and MDK as abundantly secreted, tumor-derived immunosuppressive factors which may hamper cytotoxic function of T/NK cells inside and outside the TME.

### 3.3 MIF depletion increases CAR-T cell activation

**Fig. 3:**
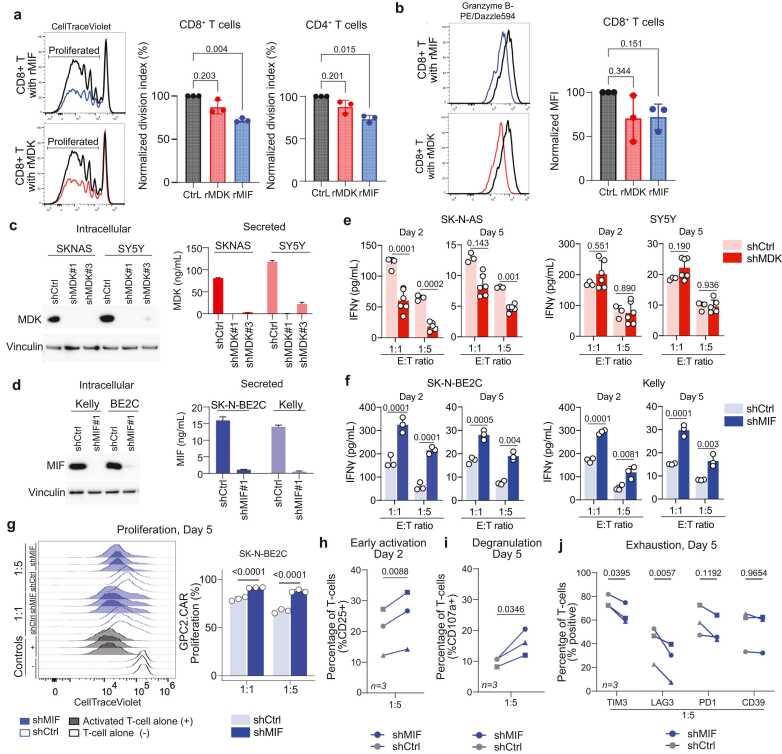
The immunosuppressive effect of MIF and MDK on CAR-T cell activation. a, Flow cytometry analysis of healthy donor peripheral blood T cells after 4 day *in vitro* stimulation with anti-CD3/anti-CD28 beads, in the presence of rMIF or rMDK (10 ng/mL). Representative graphs of CellTrace Violet peaks in CD8+ population (left panel). Middle and right panel represent the normalized division index of the CD8+ and CD4+ population from three healthy donors, respectively. Division index was determined using FlowJo’s proliferation platform. *Dunnett’s multiple comparisons test for one-way ANOVA* (*n = 3 healthy donors,* blue indicates recombinant MIF, red indicates recombinant MDK). b, Granzyme B MFI (Median Fluorescence Intensity) analysis from same experiment as Fig. 3a. Representative graphs (left two panels) of granzyme B expression in CD8+ population. Combined data from three healthy donors (right panel). MFI was normalized to stimulated control. *Dunnett’s multiple comparisons test for one-way ANOVA* (*n = 3 healthy donors,* blue indicates recombinant MIF, red indicates recombinant MDK). c, Validation of MDK knock-down by western blot (intracellular; *left*) and ELISA (secreted; *right*) on SK-N-AS and SH-SY5Y. d, Validation of MIF knock-down by western blot (intracellular; *left*) and ELISA (secreted; *right*) on SK-N-BE2C and Kelly. e, Activation of GPC2 CAR T-cells after co-culture with shCtrl and shMDK SK-N-AS and SH-SY5Y models measured by IFN-γ ELISA using two effector:target ratios (E:T; 1:1 and 1:5) at 2 different timepoints (days 2 and 5). *Two-way ANOVA with Šídák's multiple comparisons test.* (*n = 1* CAR donor with 3 technical replicates). f, Activation of GPC2 CAR T-cells after co-culture with shCtrl and shMIF SK-N-Be2C and Kelly models measured by IFN-γ ELISA using 1:1 and 1:5 E:T ratios at days 2 and 5. *Two-way ANOVA with Šídák's multiple comparisons test.* (*n = 1* CAR donor with 3 technical replicates). g, Proliferation of GPC2 CAR T-cells in co-culture with SK-N-BE2C-shCTRL (grey) or SK-N-BE2C (blue) in 1:1 and 1:5 Effector:Target ratio. Left plot show proliferation (CFSE) plots of one representative assay. *Two-way ANOVA with Šídák's multiple comparisons test.* (*n = 1* CAR donor with several technical replicates). h, % of CD25 + population in GPC2 CAR-T cells in co-culture with SK-N-BE2C-shCTRL (grey) or SK-N-BE2C (blue) in 1:5 Effector:Target ratio. Each CAR-T cell donor is connected by a line. *Statistics represent a multiple paired t-test with False Discovery Rate correction. n = 3 CAR-T cell donors*. i, % of CD107a+ population in GPC2 CAR-T cells in. Each CAR-T cell donor is connected by a line. *Statistics represent a multiple paired t-test with False Discovery Rate correction.* n = 3 CAR-T cell donors. j, Mean % of TIM3, LAG3, PD-1 and CD39 on GPC2 CAR-T cells day 5 of co-culture with SK-N-BE2C-shCTRL (grey) or SK-N-BE2C (blue). *Statistics represent 2-way ANOVA with Šídák's multiple comparisons correction n = 3 CAR-T cell donors.*

Since CAR T-cell therapy is a promising upcoming treatment strategy for neuroblastoma, we investigated the suppressive effect of MIF and MDK on T cells specifically. Healthy donor T cells were activated with anti-CD3/CD28 stimulation in the presence of recombinant MIF or MDK. We observed a significant decrease in division index when T cells were co-incubated with MIF, both in the CD8+ and the CD4+ subset, while MDK only slightly decreased proliferation (Fig. 3a, Supplementary Fig. 4a). In addition, the presence of recombinant MIF or MDK modestly decreased the cytotoxicity of CD8+ T cells, as measured by granzyme B expression, albeit not statistically significant (Fig. 3b, Supplementary Fig. 4b).

To investigate the roles of MIF and MDK on CAR T-cell modulation, we used CAR T-cells targeting GPC2, which have shown robust safety and efficacy in diverse preclinical models of neuroblastoma and are currently being tested clinically (NCT05650749) [6], [39]. To study this interaction, we generated neuroblastoma cell lines with genetic depletion of MDK (SK-N-AS-shMDK and SH-SY5Y-shMDK) or MIF (SK-N-BE2C-shMIF and Kelly-shMIF) using shRNA-mediated silencing (Fig. 3c,d, Supplementary Fig. 5a–c). We selected models with low expression of GPC2, where only moderate killing by GPC2 CAR-T cells was expected, leaving a potential window to enhance killing efficacy. GPC2 expression levels remained similar for the knock-down model to the control (Supplementary Fig. 5d). Unexpectedly, reduced MDK secretion decreased CAR T-cell activation and proliferation in co-culture with SK-N-AS, while inducing no difference in activation and proliferation in co-culture with SH-SY5Y cells (Fig. 3e, Supplementary Fig. 5e). These findings suggest that MDK may not have the highest potential as therapeutic target. Decreased MIF secretion in SK-N-BE2C did however result in increased CAR T-cell activation in two out of three CAR T-cell donors, measured by IFN-γ secretion and proliferation of CAR T-cells (Fig. 3f,g, Supplementary Fig. 5f,g). Also, in the second MIF-knock down model, we observed an increase in IFN-γ secretion and proliferation of GPC2 CAR-T cells (Fig. 3f, Supplementary Fig. 5h).

When we further explored T cell dynamics in the absence or presence of MIF, we observed an increase in early activation (CD25) in GPC2 CAR-T cells from three different T cell donors in co-cultures with SK-N-BE2C-shMIF (Fig. 3h). No difference in early activation marker CD69 was observed (Supplementary Fig. 5i). In addition, after 5 days of co-culture, we observed an increased CD107a expression, indicative of tumor specific cytotoxicity [40] (Fig. 3i). Moreover, the expression of two out of four exhaustion markers measured (LAG3 and TIM3) was decreased significantly on GPC2 CAR T-cells in co-culture with MIF knock-down cells (Fig. 3j). These data support our hypothesis that MIF suppresses T cell activation. Taken together, these mild, but significant effects support a clear functional role for MIF in modulating CAR T-cell dynamics in the neuroblastoma TME, while the more complex role of MDK on T cell function remains unclear

### 3.4 Tumor-derived MIF reduces CAR-T cell cytotoxicity *in vitro* and *in vivo*

**Fig. 4:**
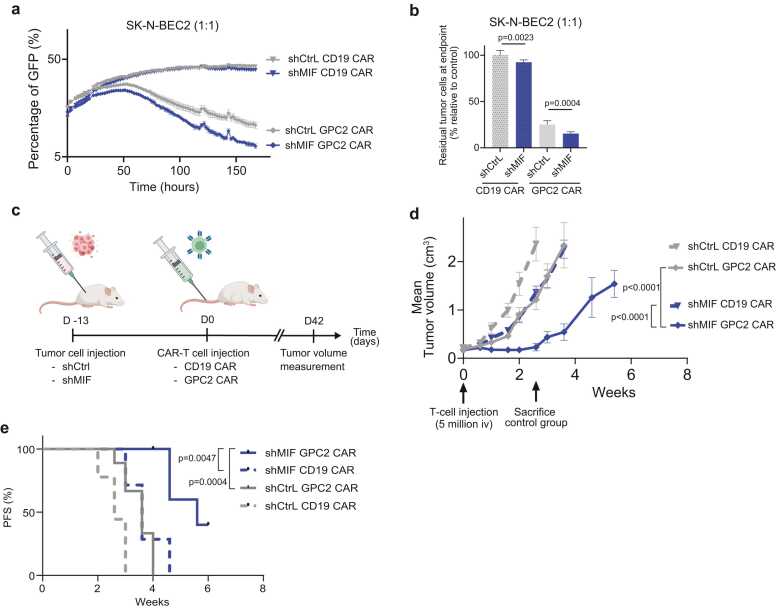
Reducing MIF secretion by the tumor increases the cytotoxicity of CAR-T cells. a, IncuCyte S3 experiment measuring tumor growth during co-culture with CAR-T cells. SK-N-BE2C neuroblastoma cells with shCtrl (grey) or shMIF (blue) were cultured with a control CAR-T cell targeting CD19 (triangles) or tumor antigen GPC2 (diamonds). Effector:Target ratio of 1:1. b, Normalized residual tumor cells at endpoint of the experiment in Fig. 4a. *One-way ANOVA statistical test with Holm-Šídák's multiple comparisons test for significance*. **c,** Schematic overview of procedure to study the effect of MIF-knock down on efficacy of CAR-T cells in mice. Figure made with Biorender.com. d, Average SK-N-BE2C shCtrl or shMIF tumor growth. Measuring of tumor size started when CD19 or GPC2 CAR-T cells were injected (5 × 10^6^, iv, at arrow indication). Experimental groups of n = 6–9. *Statistics show two-way ANOVA using Tukey’s multiple comparisons test at t = 2.6 weeks*, *when mice in the control group were sacrificed*. e, Progression free survival (PFS) of shCtrl or shMIF tumor bearing mice treated with CD19.CAR or GPC2.CAR. *Statistical analysis shows p-values for a log-rank (Mantel-Cox) test.*

To evaluate the effect of MIF on tumor cell killing by GPC2 CAR-T cells, we quantified CAR-T cell killing capacity against MIF-deficient neuroblastoma cells (SK-N-BE2C-shMIF) in co-cultures. *In vitro,* shMIF tumor cells were killed significantly more efficiently than control SK-N-BE2C cells (Figure 4a,b, Supplementary Fig. 6a,b). To validate these results *in vivo*, SK-N-BE2C-shMIF or shCtrl neuroblastoma tumors were xenografted into NSG mice. After flank tumor establishment, mice were treated with an intravenous dose of GPC2 CAR T-cells, or CD19 CAR T-cells as negative control (Fig. 4c). GPC2 CAR-T cells controlled MIF-deficient tumors more effectively than MIF-proficient tumors, leading to a significantly slower outgrowth of the tumors (Fig. 4d, Supplementary Fig. 6c), and significantly prolonged survival (Fig. 4e). Intriguingly, MIF depletion alone reduced the baseline growth rate of the tumors, possibly indicating an intrinsic dependency of neuroblastoma cells on MIF for proliferation and survival (Fig. 4b,d), which renders MIF a particularly promising target for therapeutic intervention. Taken together, these results suggest that reduction of tumor-derived MIF secretion enhances CAR-T cell efficacy both *in vitro* and *in vivo*.

### 3.5 MIF-degrading PROTAC enhances CAR T-cell activation and cytotoxicity against neuroblastoma

**Fig. 5:**
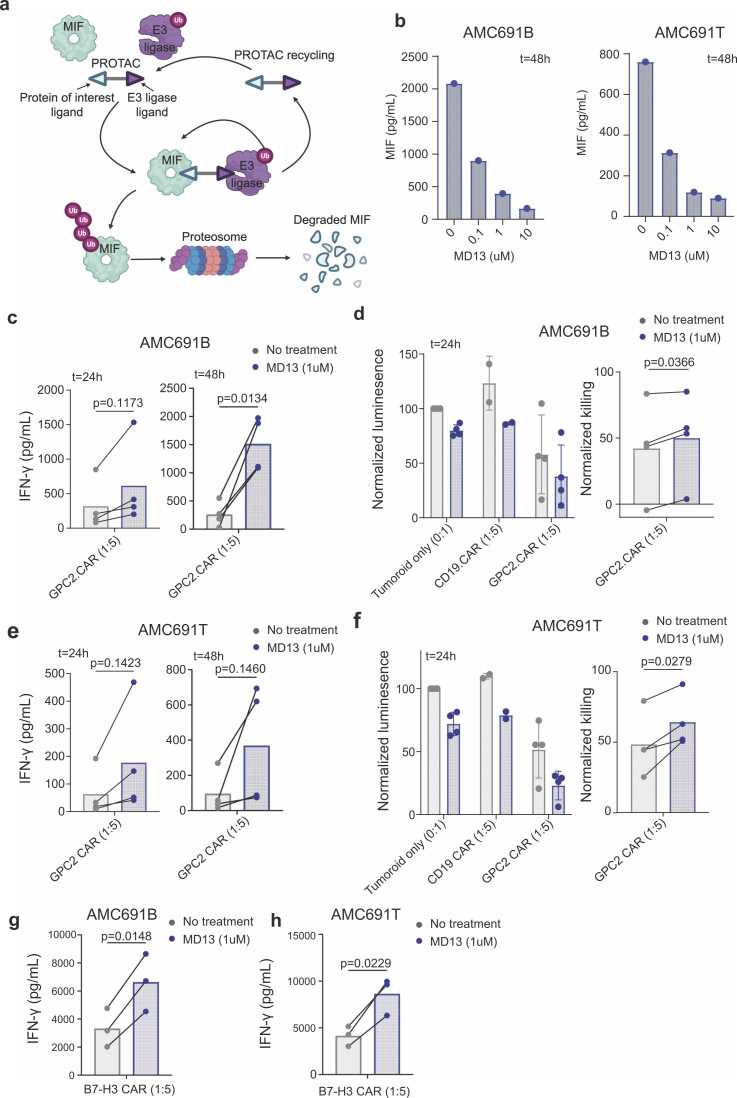
MIF PROTAC reduces MIF secretion, enabling more efficient activation of CAR-T cells. a, Schematic representation of PROTAC degraders, which causes ubiquitination and degradation of proteins of interest through the proteosome. Figure made with Biorender.com. b, MIF concentration after treatment with 0.1, 1, or 10 µM MD13 for 48 h. Left panel represents MIF concentration of tumoroid model AMC691B and right panel of tumoroid model AMC691T. Measured using Luminex. c, IFN-γ concentration of supernatant from co-culture of AMC691B without treatment (grey) or with 1uM MD13 treatment (blue) in combination with GPC2 CAR-T cell in a 1:5 Effector:Target ratio, as measured by ELISA. Left panel shows concentration at 24 h and right panel shows concentration at 48 h. *Statistical analysis shows results for paired t-test.* (*n = 4* CAR T-cell donors). d, Left panel: Luminescence signal of luciferase transduced tumoroid model AMC691B after co-culture of 24 h. Normalized to untreated tumoroid only. Tumoroids were pre-treated with or without PROTAC for 48 h before co-culture. (*n = 2* CD19-CAR T-cell donors, *n = 4* GPC2-CAR T-cell donors). Right panel: Normalized GPC2 CAR-T cell killing. Data were normalized to the tumoroid only untreated or treated control, respectively. *Statistical analysis shows results for paired t-test*. e, IFN-γ concentration of supernatant from co-culture of AMC691T without treatment (grey) or with 1uM MD13 treatment (blue) in combination with GPC2 CAR-T cell in a 1:5 Effector:Target ratio, as measured by ELISA. Left panel shows concentration at 24 h and right panel shows concentration at 48 h. *Statistical analysis shows results for paired t-test.* (*n = 4* CAR T-cell donors). f, Left panel: Luminescence signal of luciferase transduced tumoroid model AMC691T after co-culture of 24 h. Normalized to untreated tumoroid only. Tumoroids were pre-treated with or without PROTAC for 48 h before co-culture. (*n = 2* CD19-CAR T-cell donors, *n = 4* GPC2-CAR T-cell donors). Right panel: Normalized GPC2 CAR-T cell killing. Data were normalized to the tumoroid only untreated or treated control, respectively. *Statistical analysis shows results for paired t-test*. g, IFN-γ concentration of supernatant from co-culture of AMC691B without treatment (grey) or with 1µM MD13 treatment (blue) in combination with B7-H3 CAR-T cell in a 1:5 Effector:Target ratio, as measured by ELISA. (*n = 3* B7-H3-CAR T-cell donors). h, IFN-γ concentration of supernatant from co-culture of AMC691T without treatment (grey) or with 1µM MD13 treatment (blue) in combination with B7-H3 CAR-T cell in a 1:5 Effector:Target ratio, as measured by ELISA. (*n = 3* B7-H3-CAR T-cell donors).

To translate our findings towards a clinically applicable strategy, we explored the use of an innovative MIF targeting PROteolysis Targeting Chimera (PROTAC) protein degrader. PROTAC constitutes a novel therapeutic modality using the target cell’s protein degradation machinery to eliminate proteins of interest (Fig. 5a) [41], [42].

(a) Figure adapted from Dale et al. [42] .

To assess whether MIF degradation by PROTAC would reduce MIF secretion, we treated a patient-derived tumoroid model with high baseline MIF secretion (AMC691B) and a tumoroid with lower MIF secretion (AMC691T) with MD13, a PROTAC targeting MIF [43]. Indeed, treatment with MD13 reduced the concentration of secreted MIF in the culture supernatant of neuroblastoma tumoroids, with a maximal reduction of 81–84 % after 48 h (Fig. 5b, Supplementary Fig. 7a). We next combined MD13 treatment with CAR T-cells targeting GPC2, a target expressed by both tumoroids (Supplementary Fig. 7b). The tumoroids were previously transduced with a constitutively active GFP/Luciferase construct for bio-luminescence based viability assays [44]. In co-cultures, tumoroids were pre-treated with MD13 for 48 h to reduce MIF secretion before adding CAR T-cells. In line with our previous indications, GPC2 CAR T-cells were significantly more activated after 48 h co-cultures with MD13-treated tumoroids, showing a 5.8-fold increase in IFNγ production (Fig. 5c). This indicates that, indeed, MIF secreted by tumor cells suppresses CAR T-cell activation.

Similar to our observations *in vivo,* MD13 treatment alone reduced the viability of tumoroids by ∼ 20 % after 24 h (Fig. 5d). Addition of GPC2 CAR-T cells further reduced tumoroid viability to 37 %, which, when normalized to the respective (MD13-treated or untreated) ‘tumoroid only’ controls, resulted in significantly increased killing of MD13-treated tumoroids (Fig. 5d). After 48 h, only a trend towards increased killing was observed, as nearly all tumor cells were already killed even without MD13 treatment (Supplementary Fig. 7c). The second tumoroid (AMC691T), despite having lower endogenous MIF secretion, provided similar results, with significantly increased killing by CAR-T cells after 24 h of co-culture and significantly increased IFNγ production (Fig. 5e,f, Supplementary Fig. 7d).

Lastly, we confirmed our observations with a second CAR T-cell therapy targeting B7-H3, a target which was also expressed by both tumoroid models (Supplementary Fig. 7e). B7-H3 CAR T-cells were significantly more activated against MD13-treated tumoroids, as measured by IFN-γ secretion, which resulted in increased killing of the tumoroids (Fig. 5g,h, Supplementary Fig. 7f,g). These data confirm that MIF degradation results in increased activation of neuroblastoma-targeting CAR T-cells. Taken together, MIF PROTAC treatment provides a promising therapeutic strategy to reduce MIF secretion by neuroblastoma tumors and thereby enhance activation of clinically relevant CAR T-cells.

## 4 Discussion

CAR T-cell therapy is a promising new approach for the treatment of cancer. However, in solid tumors, the immunosuppressive TME remains a major challenge for CAR T-cell persistence and durability. Here, we used a multi-omics approach to pinpoint immunosuppressive factors hampering CAR-T efficacy, which identified MIF as a neuroblastoma-derived, abundantly secreted, immunosuppressive factor. Degradation of MIF by PROTAC treatment enhanced the efficacy of multiple neuroblastoma-targeting CAR T-cells, which provides a novel therapeutic strategy to enhance CAR-T cell efficacy in patients with neuroblastoma.

We observed that neuroblastoma tumor cells express high levels of MIF and partially depend on MIF for their proliferation and/or survival, which is consistent with prior findings [45]. Interestingly, MIF was initially described as a lymphocyte-secreted factor influencing macrophage motility but was later recognized as a multi-functional cytokine with context-dependent functions [46], [47], [48]. In our datasets, MIF was primarily expressed by tumor cells and less so by others cells in the TME. More recently, MIF has been described in several malignancies, including neuroblastoma, to correlate with tumor growth, metastases and survival [36], [49], [50]. MIF upregulation in neuroblastoma can induce MYCN expression, resulting in tumor progression [51]. Moreover, MIF contributes to survival and invasion of tumor cells as well as an immunosuppressive TME in bone marrow metastases [52]. Taken together, these studies suggest an important intrinsic role for MIF in neuroblastoma growth and progression, which makes MIF a particularly attractive target for therapeutic intervention.

Next to these tumor-intrinsic effects, we found that tumor-secreted MIF is associated with reduced cytotoxicity of tumor-infiltrating lymphocytes in neuroblastoma. Similar observations have been made in other malignancies. In soft-tissue sarcoma, tumor-secreted MIF shaped macrophages to become more pro-tumorigenic, and in melanoma, MIF tuned myeloid-derived suppressor cells to hinder cytotoxicity of T cells [53]. In rhabdomyosarcoma, MIF knock-out increased the potential of cellular immune interventions [54]. In addition, a recent study presenting resistance mechanisms of BCMA CAR T-cell therapy in multiple myeloma, revealed MIF as one of the major immunosuppressive signaling mediators [55]. In gastric cancer and head and neck squamous cell carcinoma patients, MIF levels were elevated in the plasma from patients with accelerated cancer progression, and plasma-MIF concentration correlated negatively with response to immune-checkpoint inhibition [56], [57]. Therefore, it is feasible that increased circulating MIF levels may even impact CAR T-cells systemically. Moreover, MIF might serve as a biomarker for patient stratification in future studies, to tailor treatment approaches. These studies corroborate our findings of an immunosuppressive effect of MIF on CAR T-cells and highlight the broad relevance and applicability of MIF-targeting therapies to enhance CAR-T cell efficacy in solid tumors.

While the direct effect of MIF on T cells and especially CAR T-cells has been scarcely studied, in our scRNA-seq analysis MIF was predicted to interact directly with T cells through the CD74 receptor, the invariant chain of major histocompatibility complex II (MHC-II). Binding of MIF to CD74 induces heterodimerization with CD44 and subsequent ERK-MAPK pathway activation, resulting in production of, amongst other factors, prostaglandin E2 (PGE2), a well-characterized suppressor of T cell activation [34], [58]. MIF can also cause activation-induced T cell death through the IFN-γ pathway [59]. Furthermore, in addition to direct effects, MIF can indirectly affect T cell function through other cells in the TME, such as by activating myeloid-derived suppressor cells or M2-like macrophages. These additional indirect functions of MIF on T cells may have contributed to the more robust effects of MIF depletion *in vivo* compared to the more moderate effects *in vitro* which we observed [60]. However, studies with more complex models are needed to confirm this.

A unique aspect of our study is our multi-omics approach, which validated MIF as a target at the RNA, protein, and secretome level, in large patient cohorts. In addition, with our highly translational pipeline we were able to validate *in silico* findings *in vitro* and *in vivo* using clinically-relevant CAR T-cells. Moreover, PROTAC-mediated MIF degradation provides a potential novel therapeutic intervention to improve CAR T-cell efficacy. While we validated the immunosuppressive effect of MIF on T cells, we could not confirm a similar effect of MDK, despite its potential as a target identified in the transcriptomics and proteomics analyses. The lack of an effect may be due to a more complex, indirect role of MDK in immunosuppression, as studies suggest it rewires the tumor microenvironment (TME) by modulating the myeloid compartment [61], [62]. Furthermore, in our interaction analyses, MDK mainly interacted with NK cells, also suggesting that the effect on T cells might be more indirect. Here, our *in vitro* and *in vivo* models assessed direct effects on T cell activation, which may explain the lack of observed impact from MDK knockdown. Further research using more complex models with additional immune cell types is needed to clarify the role of MDK in the neuroblastoma TME and its potential as a target for enhancing immunotherapy.

We are currently investigating the clinical suitability, including safety profile and efficacy *in vivo,* of MD13 and additional next-generation MIF-PROTACs to evaluate whether treatment of patients with a MIF-PROTAC before CAR T-cell administration may enhance the efficacy of the CAR T-cell product. Additionally, other interventions against MIF in clinical development may be eligible for a combination strategy with CAR-T cells. Several monoclonal antibodies targeting MIF have shown promising results *in vitro* and *in vivo,* and are being evaluated in a first clinical study in patients with solid tumors (clinicaltrials.gov; NCT01765790) [63]. Given the broad expression of MIF across solid tumors, inhibition or degradation of MIF may be a viable strategy to improve CAR T-cell efficacy not only in neuroblastoma but in many solid cancers. Moreover, our multi-omics pipeline designed to identify targetable immunosuppressive factors can be readily applied to other difficult to treat solid cancers to improve immunotherapy efficacy.

In conclusion, we have shown that selective targeting of immunosuppressive secreted factors in the TME, and MIF in particular, can improve the efficacy of CAR T-cell therapy and promote improved outcomes for patients with solid tumors.

## Funding statement

This work received funding from Villa Joep (Joining forces to activate T cell immunity against High-Risk Neuroblastoma) and Veni (Release the beast: Boosting CAR-T cell immunotherapy for neuroblastoma, 09150162010022, which is partly financed by the Dutch Research Council (NWO) and by ZonMW). In addition, this work was delivered as part of the PROTECT team supported by the Cancer Grand Challenges partnership funded by Cancer Research UK (CGCATF-2023/100050) and the 10.13039/100000054National Cancer Institute (1 OT2 CA297382). J.S. received travel grants from the Prins Bernhard Cultuurfonds and the KNAW Ter Meulen Grant for this project. G.P.P. received funding from the Alex's Lemonade Stand Foundation and The Foerderer Award from The Children Hospital of Philadelphia. K.R.B. received funding from the 10.13039/100000054National Cancer Institute (R37 CA282041 and K08 CA230223). ProCan® is supported by a National Health and Medical Research grant (GNT1170739), a companion grant to support the European Commission’s Horizon 2020 Program, H2020-SC1-DTH-2018-1, 'iPC - individualizedPaediatricCure' [ref. 826121]”. F.J.D. received funding from the Dutch Research Council (NWO) (grant no. OCENW.M.21.213). C.Z. was funded by the 10.13039/501100004543China Scholarship Council (grant no.202006220019). J.A. received funding from the Great Ormond Street NIHR Biomedical Research centre and “Research in Childhood Cancer”. T.M. and L.V. (single cell genomics facility of Princess Máxima Center for Pediatric Oncology) are funded by KiKa. W.W. is supported by Singapore Immunology Network (SIgN), Agency for Science, Technology and Research (A∗STAR); Biomedical Research Council (10.13039/100005504BMRC), Core Research Fund for use-inspired basic research (UIBR) and IAF-PP Project H22J2a0043, and Singapore National Medical Research Council (10.13039/100007680NMRC) project MOH-001401-00.

## CRediT authorship contribution statement

**Soengas María S:** Resources. **Catena Xavier:** Writing – review & editing, Resources. **Robinson Phillip:** Investigation. **Hains Peter G:** Investigation. **Schild Linda G:** Investigation. **Roger R. Redde:** Investigation. **Zhong Qing:** Investigation. **Himsworth Courtney:** Methodology, Investigation. **Barisa Marta:** Writing – review & editing, Resources, Methodology. **Muller Henrike:** Methodology, Investigation. **Shah Rivani:** Methodology, Investigation. **Visser Lindy L:** Methodology, Formal analysis. **Matlaga Stephanie:** Methodology, Investigation, Data curation. **McIntyre Brendan:** Investigation, Formal analysis, Data curation. **Kalmeijer Yannine J:** Investigation. **Wu Wei:** Writing – review & editing, Visualization, Supervision, Formal analysis, Data curation, Conceptualization. **Bosse Kristopher R:** Writing – review & editing, Supervision, Funding acquisition. **Pascual-Pasto Guillem:** Writing – review & editing, Writing – original draft, Visualization, Validation, Methodology, Investigation, Formal analysis, Data curation, Conceptualization. **Zhao Chunlong:** Resources, Methodology. **Grothusen Grant P:** Data curation, Formal analysis, Investigation, Methodology, Validation, Writing – review & editing. **Wienke Judith:** Writing – review & editing, Writing – original draft, Visualization, Supervision, Project administration, Investigation, Funding acquisition, Formal analysis, Data curation, Conceptualization. **Kalaitsidou Elisavet:** Investigation, Formal analysis. **Dekker Frank J:** Supervision, Resources. **Margaritis Thanasis:** Writing – review & editing, Formal analysis. **Molenaar Jan J:** Writing – review & editing, Supervision, Project administration, Funding acquisition. **Strijker Josephine G M:** Writing – review & editing, Writing – original draft, Visualization, Validation, Methodology, Investigation, Funding acquisition, Formal analysis, Data curation, Conceptualization. **Anderson John:** Supervision, Resources, Methodology.

## Declaration of Competing Interest

K.R.B. and G.P.P. have applied for patents for the discovery and development of immunotherapies for cancer, including patents related to GPC2-directed immunotherapies. K.R.B. receives royalties from Tmunity/Kite, a Gilead Company, and ConjugateBio, Inc. for licensing of GPC2-related technology and funding from Tmunity/Kite, a Gilead Company, for research on GPC2-directed immunotherapies. K.R.B. is on the ConjugateBio Scientific Advisory Board. M.B. holds patents pertinent to cellular immunotherapy development and manufacture, and has consulted for Lava Therapeutics. J.A. holds founder stock in Autolus ltd, consults for Roche and BMS, and holds patents in CAR-T design. J.M. has received research funding from Roche for in vitro work. R.R.R. is an advisor to Tessellate Bio and Rejuveron Telomere Therapeutics. All remaining authors have declared no conflicts of interest

## Data Availability

Single-cell RNA sequencing data for analyses in Fig. 1 were published before by our group (DOI: https://doi.org/10.1016/j.ccell.2023.12.008). Proteomics data for Fig. 2c and Supplementary Fig. 3a are available on Proteomics IDEntifications Database (PRIDE) (PXD051044 and PXD051208).

## Footnotes

Appendix A Supplementary data associated with this article can be found in the online version at doi:10.1016/j.ejca.2025.115263.
