# Supplementary material for: Blocking MIF secretion enhances CAR T-cell efficacy against neuroblastoma
Source: Eur J Cancer. 2025 Mar 11;218:None. doi: 10.1016/j.ejca.2025.115263 (PMC11884407; doi:10.1016/j.ejca.2025.115263)
Supplement: Supplementary file 1 — Supplementary material [file mmc1.pdf]

Supplementary methods

Tumoroid cultures

Tumoroids were cultured in optimized medium (DMEM, low glucose, GlutaMAX supplemented with 20% F-12 Ham's Nutrient Mix, 100U/mL penicillin, 100ug/mL streptomycin, B27 (50x), N2 (100x), hIGF (300ng/mL), hFGF (40ng/mL), hEGF (20ng/mL), PDGFaa (10ng/mL), PDGFbb (10ng/mL)). Cells were passaged 1-2 times per week by breaking up larger spheres mechanically and moving the cells to a larger flask while refreshing the culture medium. Cells were propagated at 37°C in 5% CO_2. For killing assays, tumoroids transduced with a GFP-Luciferase construct were used to be able to measure viability of the tumor cells[1].

Tumoroid secretome collection

Tumoroid cultures were expanded to two full T175 flasks. Medium was washed off and 45 mL empty DMEM GlutaMAX was left on for 24 hours to condition. The conditioned medium was centrifuged for 5 minutes at 250g remove any cells, after which the medium was centrifuged twice at 3220g for 10 minutes to remove debris. The conditioned medium was concentrated by filtering down to 1mL using a 3kDa molecular weight cut-off Millipore filter. Proteins were denatured in 4M Urea, in a Tris-buffered environment at pH 8.0. Secreted proteins were reduced in 10mM Dithiothreitol (DTT; Sigma-Aldrich) at 20°C for 1 hour, and then alkylated in 20mM iodoacetamide (IAA; Sigma-Aldrich) at 20°C for 0.5 hour in the dark. Protein digestion was performed sequentially with Lys C (1:50) and Trypsin (1:50) for 4 and 16 hours respectively. Digested peptides were acidified to 2% formic acid and diluted to 1 mL for peptide cleanup by Sep-Pak C18 1cc (Waters). Desalted peptides were dried by vacuum centrifugation, and stored at -80°C for further use.

Secretome sample preparation and LC-MS

Peptides were reconstituted with 2% formic acid and analyzed in triplicates on an Orbitrap Exploris 480 mass spectrometer (Thermo Scientific) coupled to an UltiMate 3000 UHPLC system (Thermo Scientific), that consisted of an µ-precolumn (C18 PepMap100, 5 µm, 100 Å, 5 mm × 300 µm; Thermo Scientific) and an analytical column (120 EC-C18, 2.7 µm, 50 cm × 75 µm; Agilent Poroshell). Solvent A was made of 0.1% formic acid in water and solvent B was 0.1% formic acid in 80% acetonitrile, 20% water. Peptides were resolved on a 175min gradient from 10 to 40% Solvent B. Mass spectrometry data were acquired in data-dependent acquisition mode. MS1 scans were acquired between m/z 375-1600 at a resolution of 60,000, upon signal accumulation to AGC target of 1x106. Multiply charged precursors starting from m/z 120 were selected for further fragmentation. Higher energy collision dissociation (HCD) was performed with 28% normalized collision energy (NCE), at a resolution of 30,000, upon signal accumulation to AGC target of 1e5. An isolation window of 1.4 m/z and dynamic exclusion of 24s were used.

Secretome data analysis

MS raw files were searched with MaxQuant (version 1.6.10.0) against the human UniProt database (version April 22, 2021) using the integrated Andromeda search engines. Protein N-terminal acetylation and methionine oxidation were added to variable modification, whereas cysteine carbamidomethylation was added to fixed modification. Trypsin/P was set as the enzyme for digestion and up to 2 miss cleavage was allowed. Precursor ion tolerance was set to 20 ppm for the first search and 4.5 ppm after recalibration, and fragment ions tolerance was set to 20 ppm. False discovery rate (FDR) of 1% was set at both peptide spectrum match (PSM) and protein level by using a reverse decoy database strategy. Label-free quantification (LFQ) algorithm and the match-between-run feature were enabled for protein identification. Proteins identified from empty DMEM GlutaMAX were excluded from the identifications.

Short Harpin RNA modifications

MIF or MDK knockdown was achieved using lentiviral pLKO.1_short-hairpin RNA (shRNA) constructs obtained from Sigma (TRCN0000303918, TRCN0000331210, TRCN0000331252, TRCN0000331270, TRCN0000331211, TRCN0000056818, TRCN0000056819, TRCN0000056820, TRCN0000056821 and TRCN0000056822). Lentiviral particles were produced by transfecting HEK293T cells with the shRNA plasmids and packaging plasmids, as described previously[2]. Target cells were transduced in the presence of 8 µg/mL polybrene and selected with puromycin (range of 0.5-2 µg/mL) for 7 days. Non-targeting shRNA was used as a control.

Western Blot

Protein lysates from neuroblastoma cells were separated on 4-12% Bis-Tris gels (Life Technologies), transferred to a PVDF membrane, blocked in 5% non-fat milk in Tris-buffered saline and Tween-20 (TBS-T), and blotted using standard protocols. Membranes were incubated at 4°C overnight in MIF (Cell Signaling; #87501; 1:1000), MDK (Santa Cruz; #46701; 1:200), or β-actin antibodies, washed x 3 in TBS-T and developed with a chemiluminescent reagent (SuperSignal West Femto, Thermo Fisher Scientific).

ELISA

For determining the concentration of MIF and MDK secreted by the mutants, we used Human MIF and midkine DuoSet ELISA kits (DY289 and DY258, respectively; R&D systems). Cells were plated in a 96-well plate and supernatant was collected at several time point. To account for the differences in number of cells, the luminescence signal by the GFP-luciferase construct was measured. IFN-γ secretion to validate CAR-T cell activation was measured using the Human INF-γ ELISA kit (Biolegend) or Human IFN-γ DuoSet ELISA kit (R&D Systems). Supernatant of co-cultures was harvested at indicated times and stored at -20°C until analysis.

GPC2 and CD19 CAR-T cell manufacturing

CAR constructs were generated using a lentiviral construct backbone containing an EF-1α promoter. The GPC2.CAR was designed using a CD8α leader, followed by the single-chain variable fragment (scFv) of the GPC2 D3 Barisa antibody with a VL-VH orientation with (Gly4Ser)x3 linker, a CD28 hinge and transmembrane domain, and 4-1BB and CD3-ζ co-stimulatory domains. The CD19.CAR was designed using a CD8α leader, followed by a scFv derived from the FMC63 antibody, a CD8 hinge and transmembrane domain, and 4-1BB and CD3-ζ co-stimulatory domains. DNA transfections, lentivirus production using second- and third-generation lentiviral systems, and virus transductions were performed as previously described[3].

Primary human T-cells (CD4 and CD8; 1:1 ratio) were activated for 24 hours with Dynabeads™ Human T-Expander CD3/CD28 (Thermo Fisher) beads at a 3:1 bead: T-cell ratio together with human recombinant IL-15 and IL-7 (PeproTech; 5 ng/mL each) in AIM-V medium supplemented with 5% FBS, 2mM L-Glutamine, 0.1M HEPES buffer and 1% streptomycin/penicillin at a density of $1x10^6$ cells per mL. On day 2, T-cells were transduced with CAR-containing lentiviral particles and maintained in culture until days 5-7. Then, beads were magnetically removed, and primary T-cells were placed in vented Erlenmeyer flasks at a density of $0.25x10^6$ cells per mL and cultured in agitation (125 rpm) until day 12-15. At that time, cells were collected, cell viability and CAR expression determined, and cells frozen until use in functional in vitro or in vivo assays.

Killing assays in vitro, using IncuCyte readout

CAR T-cell killing was evaluated using IncuCyte-based assays. GFP-positive, MIF wild-type or knock-down neuroblastoma cells were cultured in 96-well plates and co-incubated with CD19.41BBz or GPC2.41BBz CAR T-cells at a 1:1 effector:target (E:T) ratio. Plates were imaged every 1-2 hours using the IncuCyte ZOOM Live-Cell analysis system (Essen Bioscience). Total integrated GFP intensity per well was assessed as a quantitative measure of viable tumor cells. Values were normalized to the starting measurement and plotted over time.

Killing assay in vitro, using luminescence readout

Tumoroids transduced with a GFP-luciferase construct were cultured as described above. A single cell suspension was prepared with Accutase (Sigma Aldrich) and mechanical dissociation of the tumoroids. 10,000 single cells were plated and rested for 2-3 days to reform spheres. Effector cells were added at t=0 and left to incubate until indicated timepoints. Supernatants were collected for ELISA and D-luciferin (122799, PerkinElmer, 150ug/mL) was added to the wells and incubated for 5 minutes at 37°C. Luminescence signal was measured with the FLUOstar Omega microplate reader. All assays were performed with three technical replicates.

Flow Cytometry

Single cell suspensions were incubated with indicated antibodies and run on a Beckman CytoFLEX S or LX cytometer (Beckam Coulter). CD3-AF700,CD4-FITC, CD8-PerCP and Granzyme-B-PE/Dazzle-594 were used to stain healthy donor T cells in suppression assays. For intracellular staining of Granzyme-B, the cells were fixed using a Fix/Perm kit (Invitrogen; FOXP3). CD45-FITC or CD3-PE were used to stain T-cells in co-culture assays. GPC2 CAR expression was determined using APC or PE-conjugated human recombinant GPC2 protein, and CD19

CAR expression was determined using PE-tagged protein L. B7-H3 CAR expression was determined using CD34-APC, which binds to the co-expressed marker RQR8. For in vitro T-cell proliferation, cells were stained with CellTrace™ CFSE or Violet Cell Proliferation Kit. Data were analyzed using FlowJo software.

Supplementary Table 1: Flow Cytometry antibodies and dyes

Antigen	Fluor	Company	Cat. No.	Dilution used
CD19	PE-tagged protein L	Cell Signaling	58036S	100x
CD3	AF700	Biolegend	300324	400x
CD3	PE	Beckman Coulter	A07747	10x
CD34 Qbend10	APC	R&D Systems	FAB7227R	100x
CD4	FITC	Biolegend	357406	400x
CD45	FITC	Beckman Coulter	IM0782U	10x
CD8	PerCP	Biolegend	344707	100x
CellTrace	CFSE	ThermoFisher	C34554	2µM
CellTrace	Violet	Invitrogen	C34557	2µM
Fixable viability dye	eFluor-506	Invitrogen	65-0866-14	500x
GPC2	APC or PE conjugated recombinant GPC2	R&D Systems	2304-GP-050	500x
Granzyme-B	PE/Dazzle 594	Biolegend	372215	100x
Fixable dead cell stain	Violet-405	Invitrogen	L34964A	3,000x
CD45	APC-H7	BD Pharmingen	560178	40x
CD25	APC	BD Pharmingen	555434	10x
CD69	FITC	BD Pharmingen	555530	10x
CD107a	PE	Biolegend	328608	80x
TIM3	PE	BD Pharmingen	563422	40x
LAG3	PerCP	Invitrogen	46-2239-41	40x
CD39	FITC	BD Pharmingen	563422	40x
PD1	APC	Invitrogen	17-2799-42	40x

References:

[1] W. M. Kholosy *et al.*, ‘Neuroblastoma and DIPG organoid coculture system for personalized assessment of novel anticancer immunotherapies’, *J Pers Med*, vol. 11, no. 9, 2021, doi: 10.3390/jpm11090869.

[2] G. Pascual-Pasto *et al.*, ‘Targeting GPC2 on Intraocular and CNS Metastatic Retinoblastomas with Local and Systemic Delivery of CAR T Cells.’, *Clin Cancer Res*, vol. 30, no. 16, pp. 3578–3591, Aug. 2024, doi: 10.1158/1078-0432.CCR-24-0221.

[3] G. Pascual-Pasto *et al.*, ‘GPC2 antibody-drug conjugate reprograms the neuroblastoma immune milieu to enhance macrophage-driven therapies.’, *J Immunother Cancer*, vol. 10, no. 12, Dec. 2022, doi: 10.1136/jitc-2022-004704.
