# Supplementary material for: Blocking MIF secretion enhances CAR T-cell efficacy against neuroblastoma
Source: Eur J Cancer. 2025 Mar 11;218:None. doi: 10.1016/j.ejca.2025.115263 (PMC11884407; doi:10.1016/j.ejca.2025.115263)
Supplement: Supplementary file 2 — Supplementary material [file mmc2.pdf]

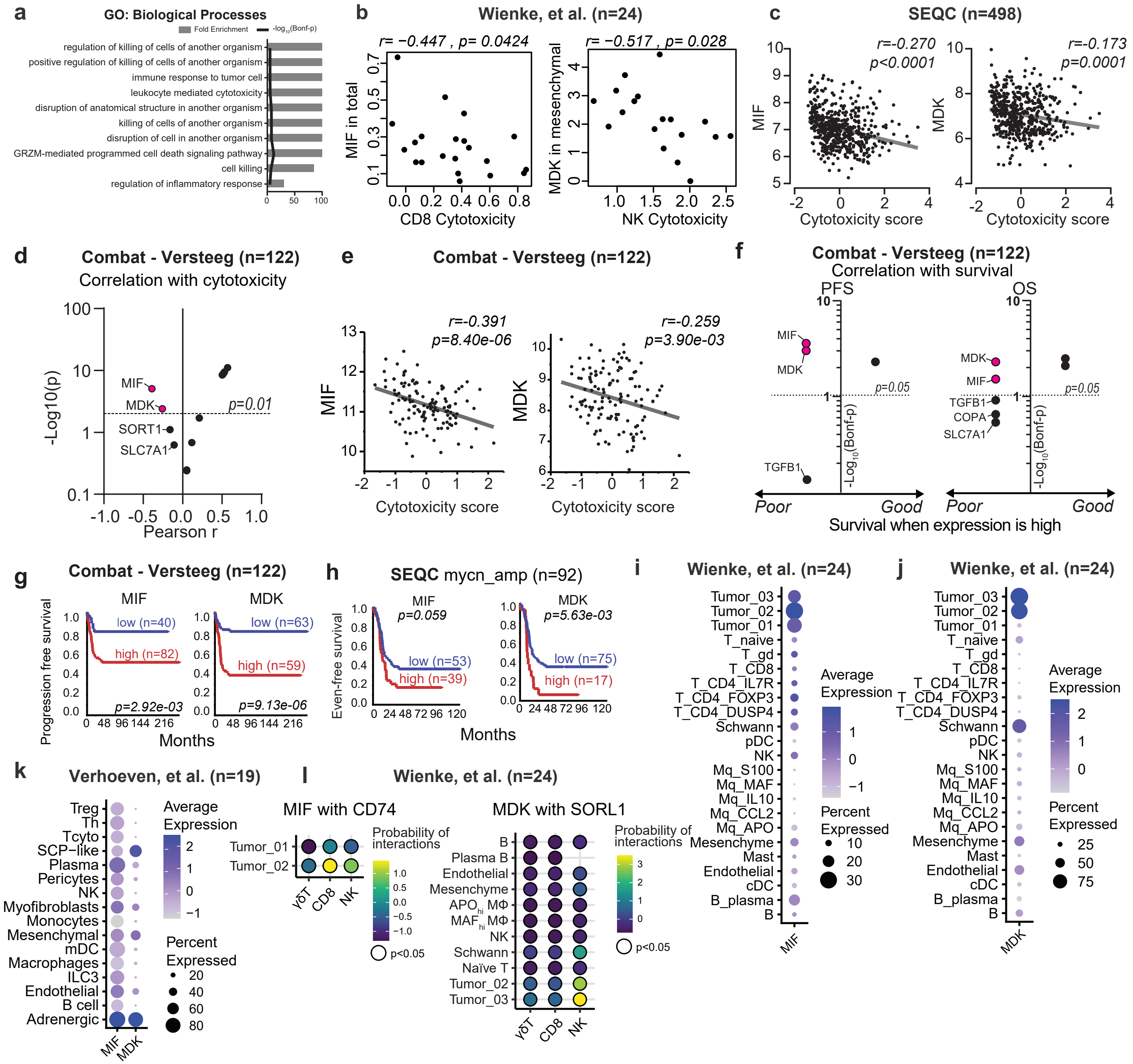

Supplementary Figure 1: a, Gene set enrichment analysis using Gene Ontology (GO) Biological Processes with the 7 selected cytotoxicity genes. Grey bar=Fold Enrichment; Black line=-log10(Bonferroni p-value). **b,** Correlation of MIF and MDK with CD8 T cell cytotoxicity and NK Cytotoxicity in scRNAseq data. **c,** Correlation of MIF and MDK with cytotoxicity score in bulk-RNAseq dataset of SEQC cohort consisting of 498 neuroblastoma tumors **d,** Correlation of 13 selected genes from Fig. 1d with cytotoxicity in dataset with bulk-RNA data from 122 neuroblastoma tumors (r2.amc.nl/; Tumor Neuroblastoma (Combat) - Versteeg - 122 - MAS5.0(bc) - u133p2; GSE16476). **e,** Correlation of MIF and MDK with cytotoxicity score in bulk-RNAseq dataset of Combat-Versteeg cohort consisting of 122 neuroblastoma tumors. **f,** Survival analysis using the 13 selected genes from Fig. 1d using the Combat - Versteeg cohort. Left panel represents progression-free survival and right panel represents overall survival. **g,** Kaplan-Meier curve indicating event-free survival for high- or low expression of MIF (left panel, expression cutoff: 2026.1) and MDK (right panel, expression cutoff: 341.7) in Combat-Versteeg dataset. **h,** Kaplan-Meier curve indicating event-free survival for high- or low expression of MIF (left panel, expression cutoff: 206.358) and MDK (right panel, expression cutoff: 276.327) only for the MYCN-amplified subset from the SEQC dataset (n=92). **i,** Bubbelplot showing the probability of interaction between MIF on cell subsets on the y-axis and MIF receptor CD74 on immune cell subsets on the x-axis (left) and the probability of interaction between MDK on cell subsets on the y-axis and MDK receptor SORL1 on immune cell subsets on the x-axis (right). Only significant values with a p<0.05 are shown. Data from the Wienke cohort has been used. **j,** Expression of MIF on separately identified cell subsets in Wienke cohort. **k,** Expression of MDK on separately identified cell subsets in Wienke cohort. **l,** Validation of expression of MIF and MDK on cell subsets in single-cell RNAseq dataset of 19 patients published by Verhoeven, et al.

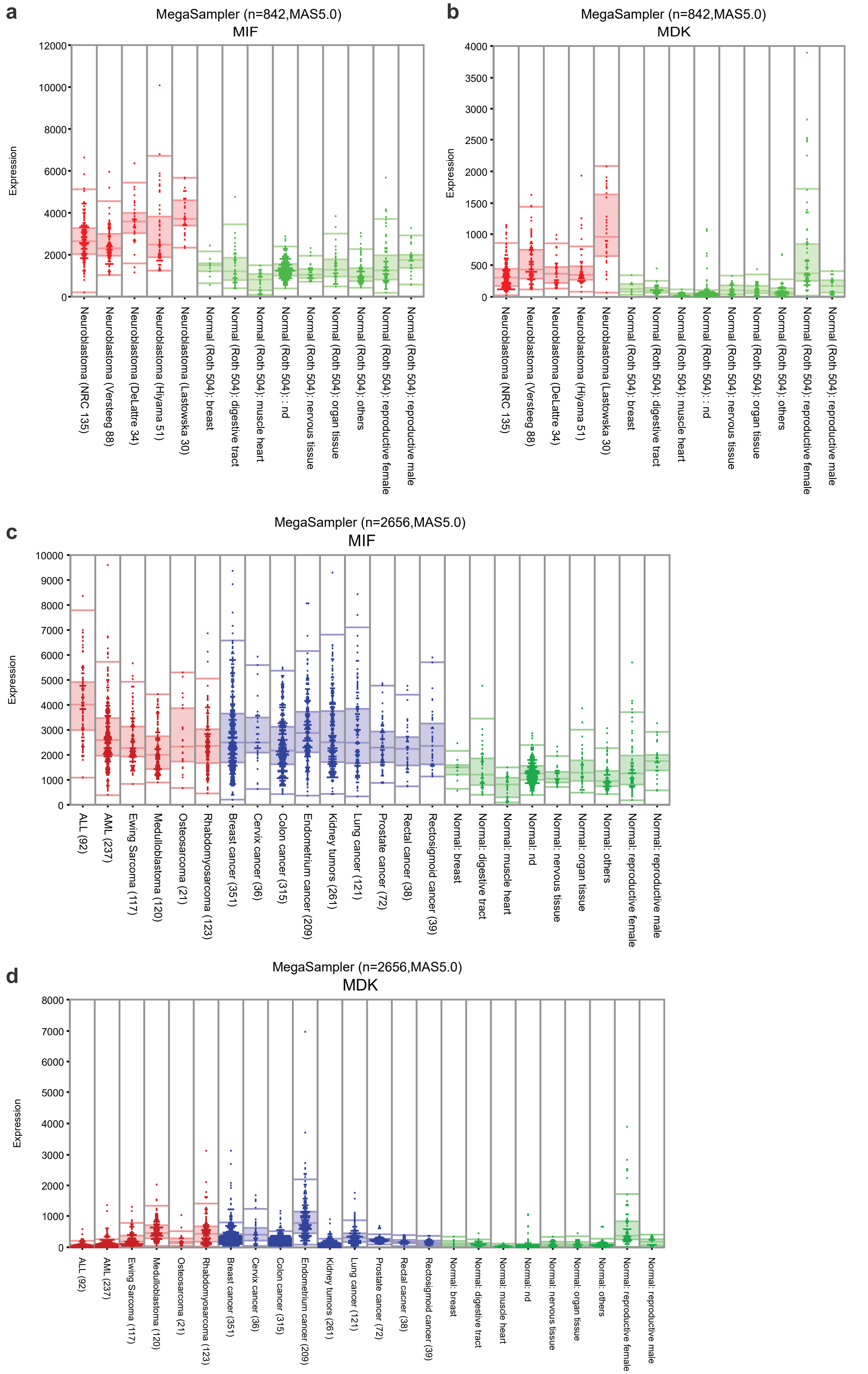

Supplementary Figure 2: a, Expression of MIF across multiple neuroblastoma (red) bulk RNAseq datasets and a healthy tissue (green) dataset with several tissue types. Figure made on r2.amc.nl with publicly available datasets. **b,** Expression of MDK across multiple neuroblastoma (red) bulk RNAseq datasets and a healthy tissue (green) dataset with several tissue types. Figure made on r2.amc.nl with publicly available datasets. **c,** Expression of MIF across multiple pediatric cancer (red), adult cancer (blue), and healthy tissue (green) bulk RNAseq datasets. Figure made on r2.amc.nl with publicly available datasets. **d,** Expression of MDK across multiple pediatric cancer (red), adult cancer (blue), and healthy tissue (green) bulk RNAseq datasets. Figure made on r2.amc.nl with publicly available datasets.

a

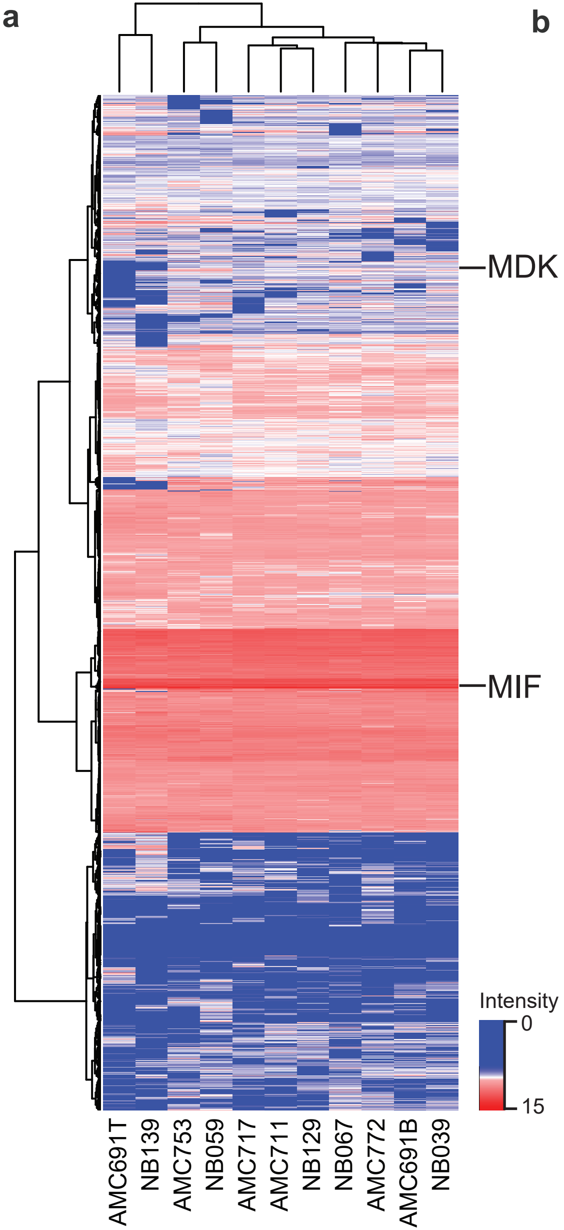

b

Tumoroid	Treatment stage	*MYCN* status	*ATRX* status	Other
NB039	Relapse	Amplified	Wild Type	1p loss, 11q loss, 17q gain
AMC691B	Diagnosis	Amplified	Wild Type	1p loss, 17q gain
AMC772	Diagnosis	Non-Amplified	Homologous deletion	11q loss, 17q gain

Supplementary Figure 3: a, Heatmap showing the expression of a total of 5,297 proteins in whole tumoroid lysates. MIF is the 20th most abundant protein with high expression in 10 out of 11 tumoroids and MDK is 3,632nd with expression in 8 out of 11 tumoroids. **b,** Table showing characteristics of three chosen tumoroids for secretome analysis.

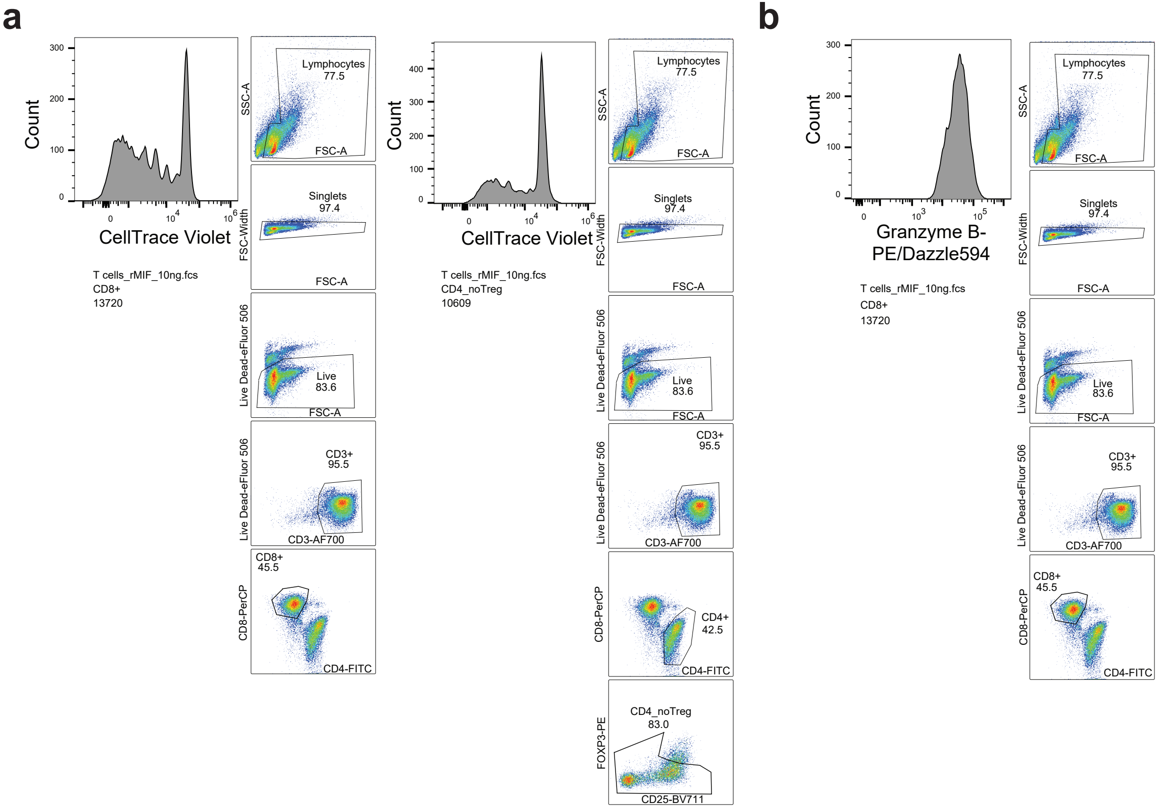

Supplementary figure 4: a, Gating strategy for Fig 3a. **b,** Gating strategy for Fig 3b.

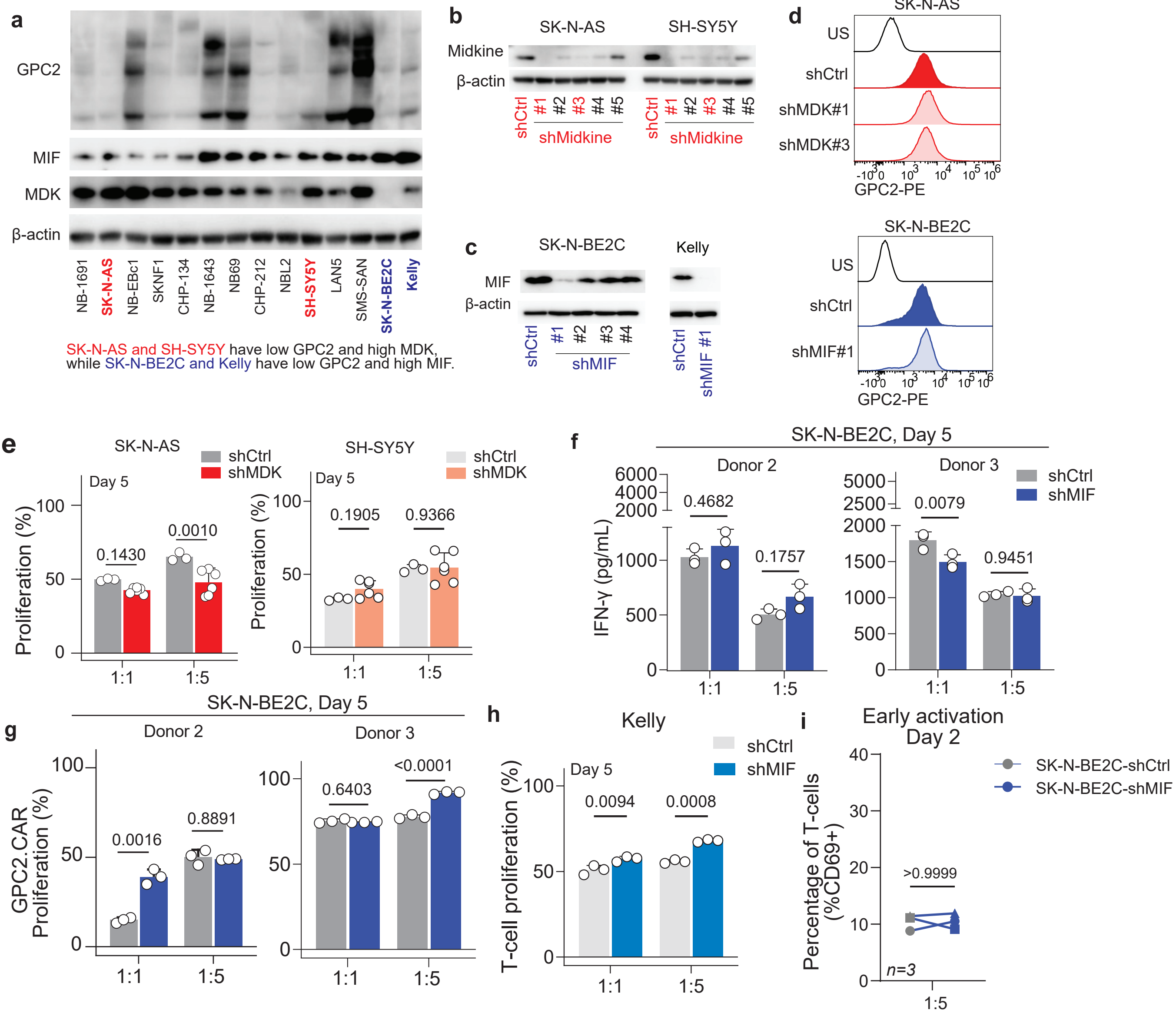

Supplementary figure 5: a, Western blot of 14 neuroblastoma cell lines for GPC2, MIF, and MDK. Two cell lines with low GPC2 and high MIF and two lines with low GPC2 and high MDK were chosen for shRNA modulation of MIF and MDK, respectively. β-actin was used as a loading control. **b,** Screening of MDK shRNAs for efficient knock-down measured by Western Blot on SK-N-AS and SH-SY5Y. shMDK constructs #1 and #3 were chosen for further testing. β-actin is used as a loading control. **c,** Screening of MIF shRNAs for efficient knock-down measured by Western Blot on SK-N-BE2C and Kelly. shMIF construct #1 was chosen for further testing. β-actin is used as a loading control. **d,** GPC2 expression by flow cytometry in shMDK and shMIF knock-down cells compared to shCtrls. **e,** Activation of GPC2 CAR T-cells in co-culture with SK-N-AS and SH-SY5Y models as measured by T cell proliferation in two effector:target ratios. shCtrl (grey) and shMDK (red). Two-way ANOVA with Šídák's multiple comparisons test. (n=1 CAR donor with several technical replicates). **f,** Activation of GPC2 CAR T-cells in co-culture with SK-N-BE2C models as measured by IFN-γ ELISA in two effector : target ratios. shCtrl SK-N-BE2C (grey) and shMIF (blue). Statistical analysis shows Two-way ANOVA with Šídák's multiple comparisons test. Each panel represents one CAR donor with three technical replicates. **g,** Activation of GPC2 CAR T-cells in co-culture with SK-N-BE2C models as measured by CAR T-cell proliferation in two effector : target ratios. shCtrl SK-N-BE2C (grey) and shMIF (blue). Statistical analysis shows Two-way ANOVA with Šídák's multiple comparisons test. Each panel represents one CAR donor with three technical replicates. **h,** Activation of GPC2 CAR T-cells in co-culture with Kelly models as measured by GPC2 CAR T-cell proliferation in two effector:target ratios. shCtrl (grey) and shMIF (blue). Statistical analysis shows Two-way ANOVA with Šídák's multiple comparisons test. (n=1 CAR donor with three technical replicates). **i,** % of CD69+ population in GPC2 CAR-T cells in co-culture with SK-N-BE2C-shCTRL (grey) or SK-N-BE2C (blue) in 1:5 Effector:Target ratio. Each CAR-T cell donor is connected by a line. Statistics represent a multiple paired t-test with False Discovery Rate correction. n=3 CAR-T cell donors.

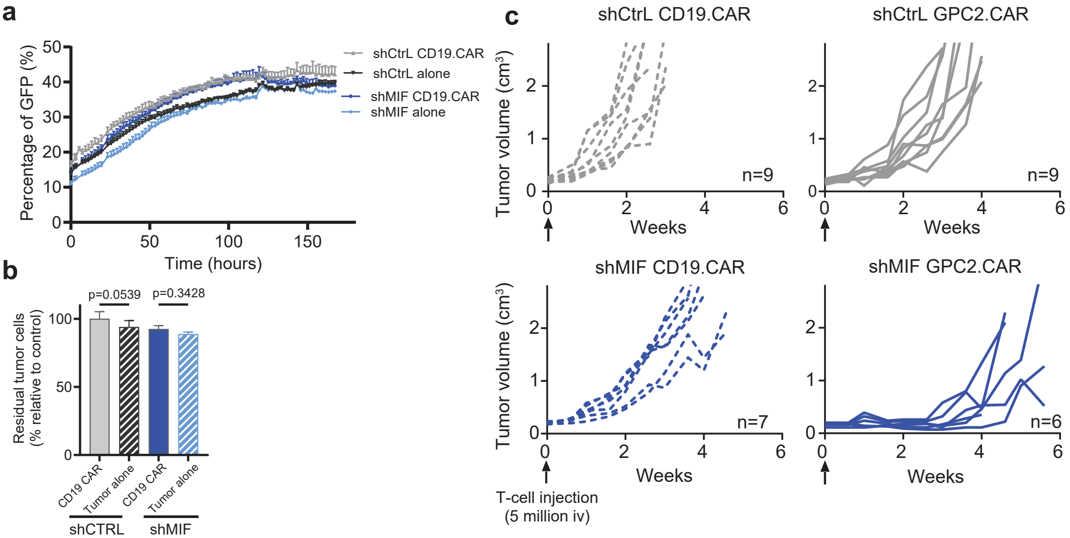

Supplementary figure 6: a, Control settings for IncuCyte S3 experiment measuring tumor growth during co-culture with CAR-T cells. SK-N-BE2C neuroblastoma cells with shCtrl (grey) or shMIF (blue) without effector cells or with CD19 CAR-T cell with Effector:Target ratio of 1:1. **b,** Normalized residual tumor cells at endpoint of the experiment in Supplementary Fig. 5a. One-way ANOVA statistical test with Tukey's multiple comparisons test for significance. **c,** SK-N-BE2C-shCtrl or shMIF tumor growth. Measuring of tumor size started when CD19 or GPC2 CAR-T cells were injected (5x106 iv, at arrow indication). Experimental groups of n=6-9.

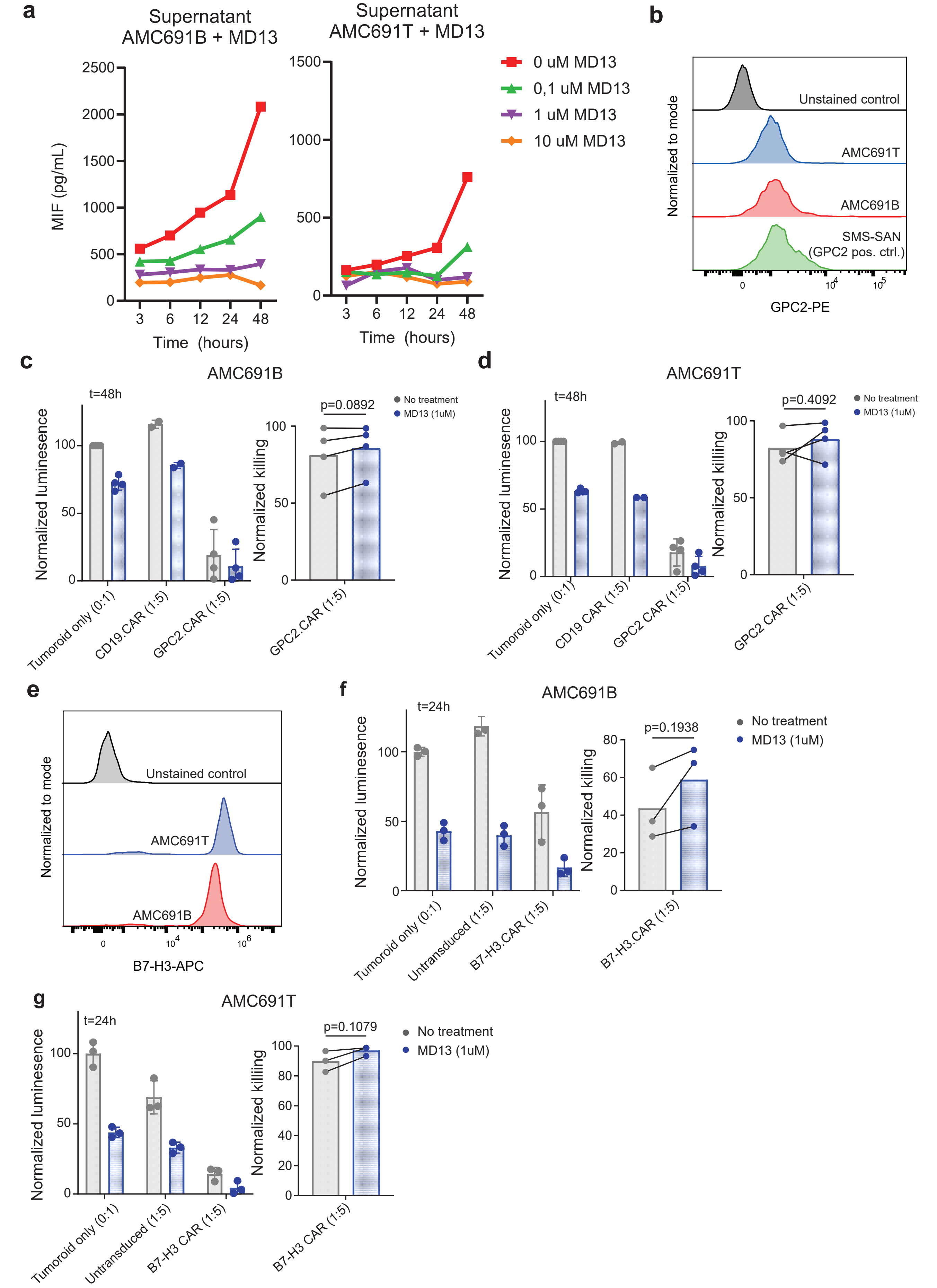

Supplementary figure 7: a, Concentration of MIF in culture supernatant after treatment with MIF PROTAC MD13. At t=0, medium was refreshed with medium containing MD13. MIF concentration was measured using Luminex. **b,** Flow cytometry results of GPC2 staining on AMC691B (red) and AMC691T (blue). SMS-SAN (green) was used as a positive control with known GPC2 expression. **c,** Left panel: Luminescence signal of luciferase transduced tumoroid model AMC691B after co-culture of 48 hours. Normalized to untreated tumoroid only. Tumoroids were pre-treated with MD13 for 48 hours before co-culture. Right panel: Normalized GPC2 CAR-T cell killing. Data were normalized to the tumoroid only untreated or treated control, respectively. Statistical analysis shows results for paired t-test. (n=2 CD19-CAR T-cell donors, n=4 GPC2-CAR T-cell donors). **d,** Left panel: Luminescence signal of luciferase transduced tumoroid model AMC691T after co-culture of 48 hours. Normalized to untreated tumoroid only. Tumoroids were pre-treated with MD13 for 48 hours before co-culture. Right panel: Normalized GPC2 CAR-T cell killing. Data were normalized to the tumoroid only untreated or treated control, respectively. Statistical analysis shows results for paired t-test. (n=2 CD19-CAR T-cell donors, n=4 GPC2-CAR T-cell donors). **e,** Flow cytometry analysis of B7-H3 staining on AMC691B and AMC691T. Unstained control is AMC691T. **f,** Left panel: Luminescence signal of AMC691B after co-culture of 24 hours. Normalized to untreated tumoroid only. Tumoroids were pre-treated with MD13 for 48 hours before co-culture. Right panel: Normalized B7-H3 CAR-T cell killing. Data were normalized to the tumoroid only untreated or treated control, respectively. Statistical analysis shows results for paired t-test. (n=3 CAR T-cell donors with untransduced controls). **g,** Left panel: Luminescence signal of AMC691T after co-culture of 24 hours. Normalized to untreated tumoroid only. Tumoroids were pre-treated with MD13 for 48 hours before co-culture. Right panel: Normalized B7-H3 CAR-T cell killing. Data were normalized to the tumoroid only untreated or treated control, respectively. Statistical analysis shows results for paired t-test. (n=3 CAR T-cell donors with untransduced controls).
